# High- versus low-anthropomorphic-cue packages and health-information trust: social presence and source credibility under explicit AI disclosure

**DOI:** 10.3389/fpubh.2026.1938022

**Published:** 2026-09-30

**Authors:** Shaohua Huo, Zhenfen Li, Yuqi Lin

**Affiliations:** Journalism and Communication School, Qilu Institute of Technology, Jinan, China

**Keywords:** AI virtual opinion leaders, bundled anthropomorphic-cue package, health information adoption intention, social presence, source credibility

## Abstract

**Background:**

AI virtual opinion leaders are increasingly used in health communication, but it remains unclear whether bundled anthropomorphic-cue packages foster initial trust and information-adoption intention when AI identity is explicitly disclosed.

**Methods:**

Guided by the Computers Are Social Actors paradigm, Social Presence Theory, Source Credibility Theory, and the Information Adoption Model, we conducted a single-factor between-subjects randomized experiment comparing overall high- and low-anthropomorphic-cue packages. The packages differed simultaneously in avatar realism, gesture behavior, interpersonal/linguistic framing, and video duration; AI disclosure and the response-button structure were held constant. A total of 536 valid responses were analyzed using partial least squares structural equation modeling. Social presence, source credibility, trust, and adoption intention were measured by participant self-report immediately after a single branched-video exposure.

**Results:**

The overall high-versus-low package contrast increased social presence. Social presence was positively associated with source credibility; source credibility was positively associated with trust in AI-generated health information; and information trust was positively associated with health information adoption intention. The direct effect of the package contrast on information trust was not significant, whereas the bootstrapped serial indirect effect through social presence and source credibility was significant.

**Conclusion:**

Within the present Chinese sample and single-exposure setting, the randomized contrast supports an effect of the overall cue package on initial social presence, while the mediation estimates are consistent with the hypothesized indirect pathway to information trust. The design cannot identify the independent contribution of any visual, gestural, linguistic, vocal, interactive, or duration component. The outcome is self-reported adoption intention, not observed health behavior.

## 1 Introduction

AI-driven virtual opinion leaders (VOLs) have expanded beyond digital marketing into health communication (1). These nonhuman agents combine a personified identity, visual presentation, language, voice, and interactive responsiveness to communicate opinions or health information (2, 3). Their use in health education, chronic disease management, and mental health communication raises a central question: can a visibly artificial communicator be perceived as socially present and credible without encouraging unwarranted trust (4, 5)? Evidence remains mixed. More realistic AI presenters can receive higher initial ratings of expertise and trustworthiness, yet AI communicators may remain less persuasive than human professionals in consequential health decisions when accountability is salient (5–7). These findings call for a more precise account of how anthropomorphic design relates to social experience, source evaluation, and information trust.

Anthropomorphic cues (AC), defined as simulations of human physical, emotional, and cognitive states, are central to understanding the persuasive mechanisms of VOLs (8, 9). This view aligns with the Computers Are Social Actors (CASA) paradigm, which posits that individuals may apply social rules to computers and respond to media cues as if they were interpersonal cues (10). In digital health communication, anthropomorphic design can function as an external stimulus that shapes the social attractiveness, interactivity, and persuasiveness of nonhuman entities (11). Chen et al. (12) found that visual and conversational anthropomorphic cues can facilitate user trust. However, any persuasive implication depends on how these technological cues are interpreted by particular audiences and should not be assumed to be uniformly positive.

In this internalization process, social presence (SP) serves as a potential bridge (13), reflecting the degree to which audiences feel that a machine entity is co-present, warm, and socially salient (14). In health contexts involving disease prevention and physiological risk, people may seek information while experiencing uncertainty about both the topic and the technology. Human-like appearance, facial expression, natural language, and emotional expression can reduce psychological distance in some human-machine interactions (15, 16). Within the tested setting, the study therefore treats anthropomorphic cues as an antecedent of participants’ initial social-presence ratings; it does not assume that the cues reduce AI anxiety for all users.

Perceived warmth and presence do not necessarily establish a VOL’s professional reliability (9). Social presence may nevertheless provide a relational basis for evaluating source credibility (SC), which Source Credibility Theory defines primarily in terms of expertise and trustworthiness (17). After exposure to anthropomorphic cues, users may evaluate whether the VOL is a believable health communicator (15). Prior research sometimes treats social presence, trust, and credibility as interchangeable, obscuring the distinction between a social experience and an assessment of competence (18). The present model therefore treats SP and SC as related but distinct post-exposure constructs.

Source evaluation and message evaluation are also distinct. Source credibility concerns perceived characteristics of the communicator, whereas trust in AI-generated health information (TAHI) concerns the perceived accuracy, reliability, and usefulness of the health information itself (19, 20). A favorable assessment of the VOL may therefore be associated with greater trust in the information it presents (6, 21). The proposed model tests SC as a statistical mediator linking SP to TAHI, while recognizing that all three constructs were measured at the same post-exposure occasion.

Ultimately, health communication may seek to support informed health decisions, but the present outcome is health information adoption intention (HIAI), not observed behavior. In this study, HIAI means that audiences report willingness to refer to the information when making health decisions, search for additional evidence, consult professionals, consider applying the recommendation, or share the information. The Information Adoption Model suggests that cognitive trust in information is associated with a greater intention to internalize or use it (22). Because intentions do not always translate into action, any relation between trust and HIAI should be interpreted separately from actual verification, clinical follow-through, or health behavior (23).

Recent research has also examined the consequences of disclosing AI authorship. Disclosure can change message evaluations and may activate algorithm aversion, while AI identity can weaken behavioral intentions in settings where accountability is important (5, 24). The present study holds explicit AI disclosure constant and asks whether an overall anthropomorphic-cue package is associated with social presence and trust-related responses under that disclosed-AI condition.

Three gaps motivate the study. First, much VOL research has examined commercial rather than health communication (2, 4). Second, the links among technological cues, social presence, source credibility, and trust in AI-generated health information remain insufficiently differentiated (11, 25). Third, anthropomorphic-cue effects have rarely been examined when AI identity is explicitly disclosed and held constant across conditions (24).

To address these theoretical gaps, this study tests whether the experimentally manipulated overall high-versus-low anthropomorphic-cue package contrast increases initial social presence after a single disclosed-AI interaction; whether post-exposure social presence and source credibility are statistically consistent with a serial indirect pathway to trust in AI-generated health information; and whether information trust is associated with health information adoption intention. AI disclosure and the platform response-button structure were held constant, so the study does not estimate the independent or interactive effect of disclosure or any single cue component. The study poses the following research questions:

*RQ1:* Under explicit AI identity disclosure, does the overall high-versus-low anthropomorphic-cue package contrast increase social presence?

*RQ2:* Are post-exposure social presence and source credibility statistically consistent with a serial indirect pathway linking the overall anthropomorphic-cue package contrast to trust in AI-generated health information?

*RQ3:* Is trust in AI-generated health information positively associated with health information adoption intention?

## 2 Theoretical underpinnings and hypotheses development

### 2.1 Theoretical underpinnings

#### 2.1.1 Computers are social actors paradigm

The Computers Are Social Actors (CASA) paradigm proposes that people can apply interpersonal rules and expectations to technological entities when systems display recognizable social cues (10). In digital health communication, CASA helps explain why visual appearance, conversational style, voice, and emotional responsiveness may shape reactions to an AI virtual opinion leader (12). Explicit AI disclosure does not remove these cues, but it may change how users interpret them (26). CASA therefore provides the starting point for examining how the tested cue package relates to participants’ social responses.

#### 2.1.2 Social presence theory

Whereas CASA explains why technological cues can elicit social responses, Social Presence Theory describes the resulting sense that another entity is present, responsive, and socially salient (14). In human-machine communication, social presence includes perceived co-presence, psychological proximity, and relational warmth. These perceptions can matter in health communication, where users may be uncertain about both the health topic and the technology providing the information (3). Social presence has therefore been used to explain trust-related responses to chatbots, virtual medical assistants, and digital health platforms (27, 28). In the present model, SP is the immediate measured response to the randomized cue-package contrast.

#### 2.1.3 Source credibility theory

Source Credibility Theory focuses on how audiences evaluate a communicator’s expertise and trustworthiness (17). This distinction is especially important in health communication, where a socially engaging source is not necessarily a professionally credible one. Users may judge whether an AI communicator appears knowledgeable, competent, honest, and objective before relying on the information it provides (1, 21). In the proposed model, SC captures this evaluation and is conceptually distinct from both SP and trust in the message.

#### 2.1.4 Information adoption model

The Information Adoption Model (IAM) evolved from the Technology Acceptance Model and the Elaboration Likelihood Model and explains how audiences evaluate and internalize information in digital environments (22). Within health communication, it is useful for examining whether audiences intend to use health information after evaluating its quality and utility. When people encounter health advice from AI virtual opinion leaders, perceived evidence quality and trust may shape willingness to consult, verify, or consider the advice. Such reported willingness is not equivalent to adherence, clinical follow-through, or actual behavior. The IAM therefore provides a framework for the proposed association between trust in AI-generated health information and HIAI, while the intention-behavior gap remains outside the present design (23).

### 2.2 Hypotheses development

#### 2.2.1 Anthropomorphic cues and social presence

In human-computer interaction design, anthropomorphic cues (AC) can endow nonliving systems with human-like visual, auditory, or interactive features (8). Following CASA, facial micro-expressions, natural speech, and responsive feedback may activate social heuristics (12). Research on virtual humans and medical chatbots has found that visual realism and humanized conversational style can increase social-presence ratings within particular designs (7, 11). By simulating aspects of interpersonal interaction, multimodal cues may make an interface feel warmer and more socially responsive. The present experiment tests this contrast only across its high- and low-cue stimuli and does not assume a monotonic benefit at untested levels. This study proposes the following hypothesis:

*H1:* Compared with the overall low-anthropomorphic-cue package, the overall high-anthropomorphic-cue package will significantly increase users’ social presence.

#### 2.2.2 Social presence and source credibility

Social presence (SP) may provide a relational basis for evaluating an AI communicator. When audiences experience stronger social presence, warmth and responsiveness may be carried into favorable judgments of the source’s competence or character (8). In health communication, this process is plausible but not deterministic: perceiving an AI as socially present does not itself establish professional competence. The present study therefore tests whether participants’ post-exposure SP ratings are positively associated with their source-credibility ratings.

*H2:* Social presence is positively associated with source credibility.

#### 2.2.3 Source credibility and trust in AI-generated health information

Once audiences evaluate the communicator, they may use that source judgment when evaluating the information itself. Trust in AI-generated health information (TAHI) reflects endorsement of the reliability and usability of the health information communicated by the VOL (29). Source Credibility Theory suggests that messages from sources perceived as expert and trustworthy are evaluated more favorably (17). In the present model, this proposition concerns the association between participants’ post-exposure evaluations of the source and the information; it does not establish causal ordering between two simultaneously measured perceptions. This study proposes the following hypothesis:

*H3:* Source credibility is positively associated with trust in AI-generated health information.

#### 2.2.4 The mediating role of social presence

Anthropomorphic cues may shape source evaluations indirectly through the social experience they evoke (18, 28). Humanlike appearance, speech, and responsiveness can make an AI communicator feel more socially present (12). That relational experience may then be associated with more favorable evaluations of the communicator’s competence and trustworthiness (2, 27). The study therefore tests SP as a statistical mediator between the cue-package contrast and SC.

*H4:* Social presence statistically mediates the relationship between anthropomorphic cues and source credibility.

#### 2.2.5 The mediating role of source credibility

Social presence alone does not establish that health information is accurate or professionally sound (20). Under explicit AI disclosure, users may separately evaluate whether the nonhuman communicator appears competent and trustworthy (27). Source credibility can therefore connect a relational response to an evaluation of the message itself. Participants who report stronger SP may also rate the source more favorably, and those source evaluations may in turn be associated with greater TAHI (6, 21). The study accordingly tests SC as a statistical mediator between SP and TAHI.

*H5:* Source credibility statistically mediates the relationship between social presence and trust in AI-generated health information.

#### 2.2.6 The chain mediation of social presence and source credibility

Taken together, the proposed relationships specify a sequence from the randomized cue-package contrast to SP, SC, and TAHI. CASA suggests that the cue package may influence the immediate social experience, while Social Presence Theory and Source Credibility Theory distinguish subsequent relational and evaluative responses (3, 12). The model therefore tests whether SP and SC form a serial indirect pathway from the cue-package contrast to TAHI (6). Because SP, SC, and TAHI were measured at one post-exposure occasion, this mediation test evaluates a theoretically ordered statistical pathway rather than fully identifying causal order.

*H6:* The indirect association between anthropomorphic cues and trust in AI-generated health information operates sequentially through social presence and source credibility.

#### 2.2.7 Trust in AI-generated health information and health information adoption intention

According to the Information adoption model, HIAI refers to a reported inclination to internalize received guidance or consider it in later decisions (22). Trust in AI-generated health information may be associated with a stronger willingness to refer to, verify, discuss, or consider that information. However, self-reported willingness cannot demonstrate that participants subsequently changed a health behavior, verified a claim, consulted a clinician, or followed medical advice. The present hypothesis is therefore limited to the relation between information trust and adoption intention (23).

*H7:* Trust in AI-generated health information is positively associated with health information adoption intention.

Based on H1–H7, the conceptual model maps the proposed relationships among the variables (Figure 1). The experimentally manipulated contrast is the effect of the overall high-versus-low anthropomorphic-cue package on initial social presence. The remaining links represent post-exposure associations and bootstrapped indirect effects among self-reported social presence, source credibility, trust, and adoption intention. This distinction is central to interpreting the model: the indirect-effect estimates are compatible with the proposed sequence but do not, by themselves, establish the causal order of simultaneously measured mediators and outcomes or the independent effect of any single cue component.

**Figure 1:**
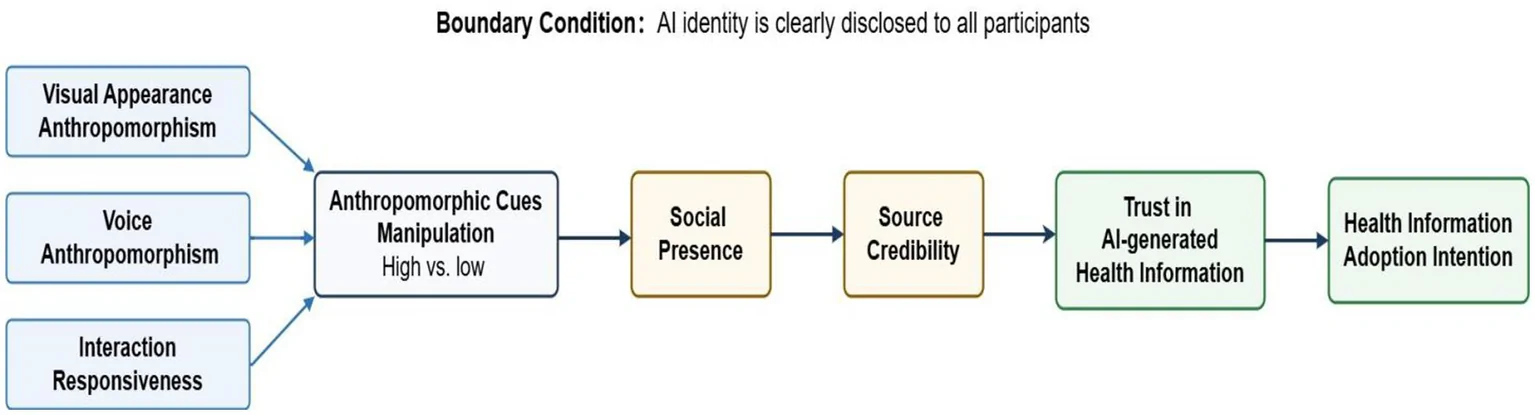
Conceptual framework. The high-versus-low cue-package contrast was randomized. SP, SC, TAHI, and HIAI were measured after exposure; their downstream paths represent theoretically structured associations and indirect effects rather than a fully established causal chain.

## 3 Materials and methods

### 3.1 Participants and sampling

Before formal data collection, an *a priori* power analysis was conducted using G*Power 3.1 (30). The analysis specified *f*^2^ = 0.10, *α* = 0.05, power = 0.95, and four predictors. Under these assumptions, the minimum required sample size was approximately 480. Allowing for invalid responses, the study planned to collect 540 raw cases to ensure sufficient statistical power after data cleaning.

Quota sampling was used during recruitment to maintain a relatively balanced gender distribution and to allocate the initially recruited sample across three age strata: 18–25 years (*N* = 180), 26–35 years (*N* = 180), and 36 years and above (*N* = 180). The upper stratum was broad and was not designed as a dedicated older-adult quota (for example, 60 or 65 years and above). Accordingly, the design does not support age-specific inference about older adults.

A total of 540 questionnaires were obtained. After responses with extremely short completion times, duplicate IP addresses, anomalous device records, or incomplete data were excluded, 536 valid cases remained. Two responses were excluded from each cue-package condition, leaving 268 valid cases in both the overall high- and low-anthropomorphic-cue packages.

### 3.2 Experimental procedure

The study adopted a single-factor between-subjects randomized human-participant experiment implemented on the Wenjuanxing interactive experiment platform. It tested how a one-session, single branched-video exposure to an overall high-versus-low anthropomorphic-cue package affected initial social presence and how post-exposure self-reports of social presence, source credibility, trust in AI-generated health information, and HIAI were related. The design did not isolate individual cue components, model repeated exposure, or test a long-term relationship with the VOL.

After entering the experiment page, participants read the study instructions and provided electronic informed consent. The system then randomly assigned them in a 1:1 ratio to either the overall high-anthropomorphic-cue package (*N* = 270) or the overall low-anthropomorphic-cue package (*N* = 270). The stimulus focused on the prevention of health risks associated with insufficient sleep, insomnia, and habitual late-night behavior. This topic was selected because sleep problems are common in everyday life and frequently discussed online. In addition, insomnia and chronic sleep deprivation involve both medical risk and actionable health advice, such as safe melatonin use, making the topic suitable for testing trust in and adoption of AI-generated health information.

Participants viewed one branched health-science video in which AI identity was disclosed. Across conditions, the core sleep-health knowledge points, number of supporting arguments, information order, recommendation content, medical disclaimer, AI disclosure, and response-button text, order, and logic were standardized. During playback on the Wenjuanxing platform, the video paused automatically at selected knowledge points and displayed two response buttons. After a participant selected an option, the corresponding prerecorded branch continued. Immediately after this single exposure, participants completed the anthropomorphic-cue manipulation check, the focal self-report measures, an attention check, and demographic questions. The manipulation-check and focal-construct items used seven-point Likert-type response scales. Figure 2 summarizes the participant-facing experimental sequence.

**Figure 2:**
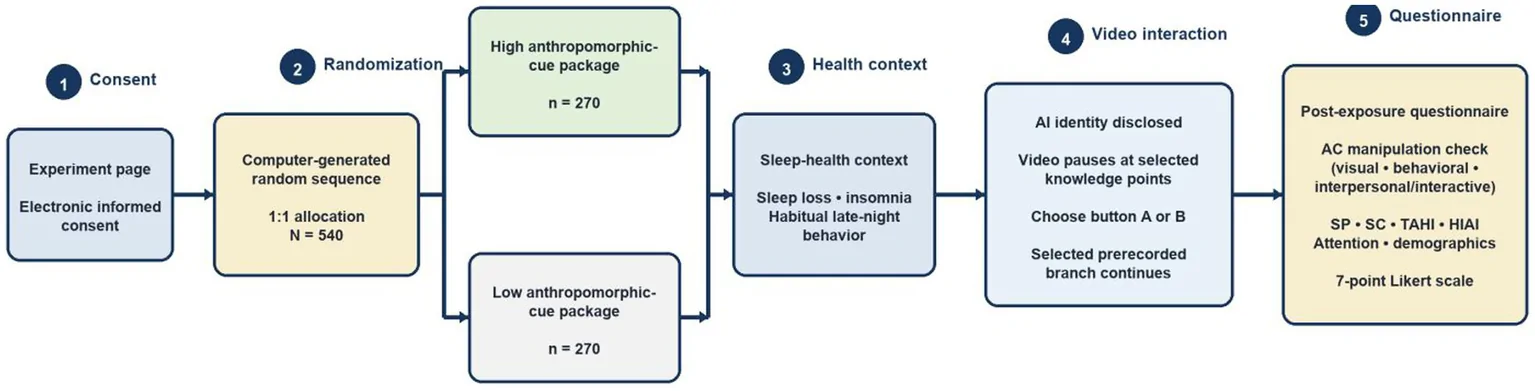
Experimental procedure. After electronic informed consent, computer-generated 1:1 randomization allocated 270 participants to each overall cue-package condition. The core sleep-health knowledge points, number of arguments, information order, recommendation content, AI disclosure, and response-button logic were standardized across conditions. At selected knowledge points, the video paused and the participant selected one of two buttons, after which the corresponding prerecorded branch continued. Immediately after exposure, participants completed the anthropomorphic-cue manipulation check covering visual, behavioral, and interpersonal/interactive perceptions; the focal constructs; an attention check; and demographic questions. Manipulation-check and focal-construct items used a seven-point scale from 1 (strongly disagree) to 7 (strongly agree).

The original stimulus videos and the recorded experiment text were rechecked condition by condition. Both presented sleep-related health information in Mandarin, used simplified-Chinese subtitles, showed the same room background, displayed an ‘AI-generated’ label, and included the medical disclaimer that the material could not replace physician diagnosis or treatment. The packages differed simultaneously in avatar realism, visible gesturing, interpersonal/linguistic framing, and duration, as summarized in Table 1 and illustrated in Figure 3. The source MP4 files were linear and did not display the Wenjuanxing response-button branches; therefore, Figure 3C is explicitly presented as a schematic reconstructed from the recorded experiment materials rather than as an original platform screenshot.

**Table 1:** Comparison of the overall high- and low-anthropomorphic-cue packages.

Feature dimension	Low-anthropomorphic-cue package	High-anthropomorphic-cue package
Visual representation	Stylized three-dimensional female avatar wearing a white blouse and dark skirt, with simplified facial realism.	More photorealistic female digital presenter wearing a light-blue dress, with more realistic facial and hair rendering.
Presentation behavior	Predominantly upright neutral stance with the arms at the sides and limited gesture variation.	Repeated two-handed and one-handed gestures, with changing hand positions and more varied presenter movement.
Linguistic/interpersonal framing	Opened directly with the AI-generated-health-information notice and medical disclaimer, followed mainly by expository health information.	Added a greeting and self-identification as an AI virtual health opinion leader and used direct-address/inclusive wording before the medical disclaimer and health information.
Audio and subtitles	Spoken Mandarin audio synchronized with simplified-Chinese subtitles.	Spoken Mandarin audio synchronized with simplified-Chinese subtitles. Voice was not isolated as an independent experimental factor.
Video duration	Approximately 150.64 s.	Approximately 178.44 s, reflecting additional introductory and conversational material.
Interactive structure	On Wenjuanxing, two standardized response buttons selected prerecorded branches; the buttons were not embedded in the source MP4.	On Wenjuanxing, the same two standardized response buttons selected prerecorded branches; the buttons were not embedded in the source MP4.
AI disclosure and disclaimer	Persistent ‘AI-generated’ label and the same medical disclaimer.	Persistent ‘AI-generated’ label and the same medical disclaimer.

**Figure 3:**
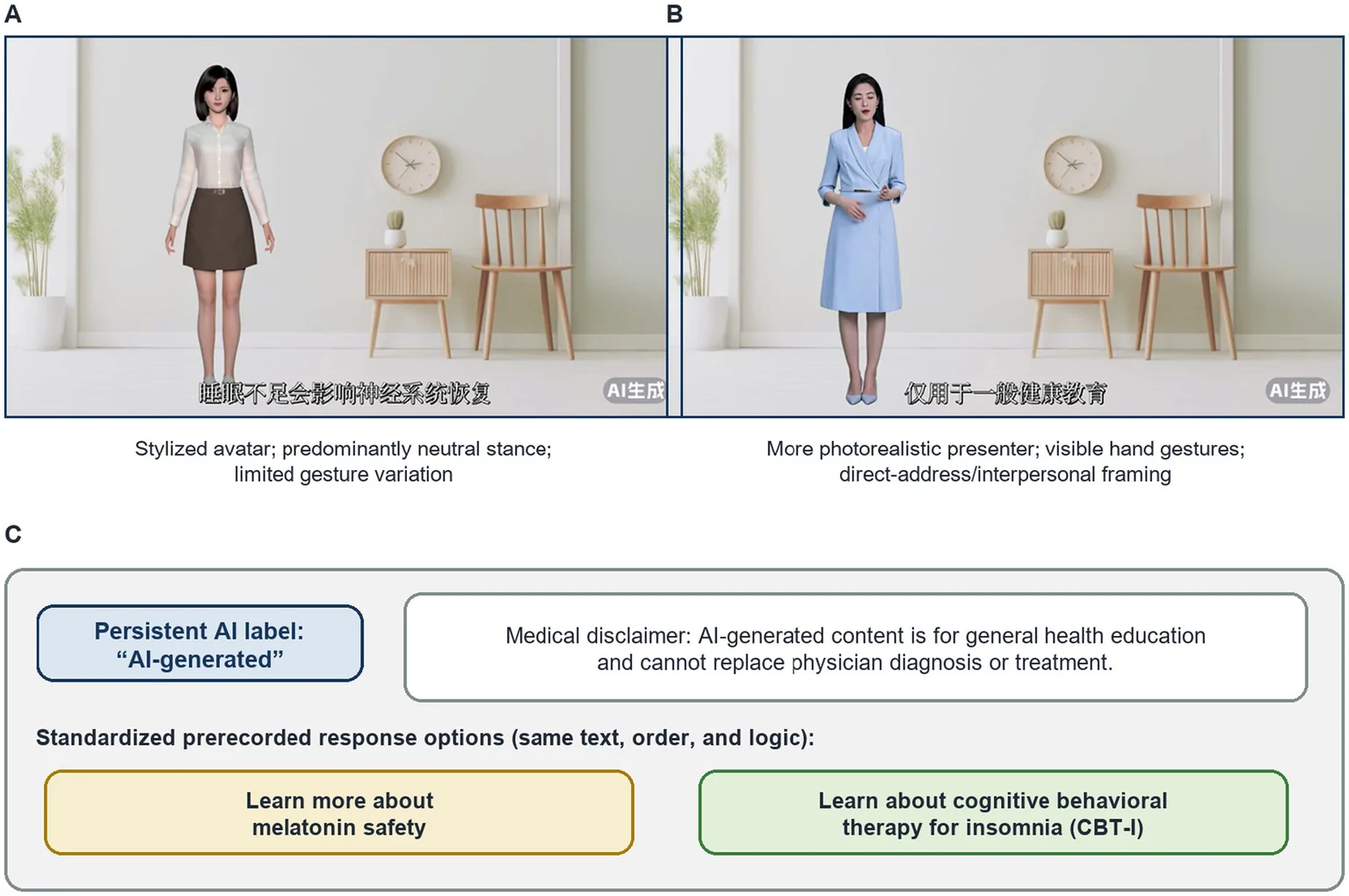
Representative experimental stimuli and common response interface. **(A)** Low anthropomorphic-cue package. **(B)** High anthropomorphic-cue package. **(C)** Interface features common to both conditions. Panels **A,B** are unaltered frames extracted at 15 seconds from the source MP4 files. Panel **C** is a schematic reconstructed from the recorded experiment materials because the Wenjuanxing response buttons were not embedded in the source MP4 files. The screenshot frames retain the original subtitles and persistent AI-generated label.

The comparison confirms that the manipulation was bundled rather than component-isolated. In addition to visual realism and presentation behavior, the high-cue package contained a greeting, explicit self-identification, direct-address/inclusive wording, and approximately 27.8 s of additional material. The experiment therefore estimates the effect of the overall high-versus-low cue package and cannot determine whether visual, linguistic, vocal, behavioral, interactive, or duration-related features independently produced the observed effect. Cue-isolation, factorial, and component-specific experiments with equivalent content and duration are needed to identify feature-specific contributions.

### 3.3 Measures

The latent variables were measured with established scales adapted to the present context (Table 2). All items used a seven-point Likert-type response scale and were completed by participants immediately after the experimental exposure.

**Table 2:** Constructs and measurement sources.

Construct	Concept	Measurement source
Anthropomorphic cues	The perceived human similarity of the AI virtual opinion leader in appearance, language expression, and interactive response.	Araujo (40) and Dabiran et al. (2)
Social presence	The audience’s perceived co-presence, interactivity, warmth, and sense that the AI virtual opinion leader resembles a real social actor.	Hassanein and Head (41)
Source credibility	The audience’s credibility evaluation of the AI virtual opinion leader as a health or science communication source, including expertise, trustworthiness, competence, and honesty.	Hendriks et al. (35)
Trust in AI-generated health information	Trust in the scientific basis, transparency, reliability, and usability of the health information communicated by the AI virtual opinion leader.	Nadelson et al. (42)
Health information adoption intention	The intention to adopt, refer to, further search for, consult professionals about, or share the health information in health decision making.	Sussman and Siegal (22)

All focal mediators and outcomes (SP, SC, TAHI, and HIAI) were obtained from the same participants by self-report at one post-exposure measurement occasion. The study did not observe platform logs, click-through behavior, information verification, clinician consultation, or subsequent health behavior.

### 3.4 Data analysis strategy

Analysis proceeded in two stages. SPSS was used for data screening, missing-data assessment, descriptive statistics, correlations, and distributional checks. Skewness and kurtosis were examined, and variance inflation factors (VIFs) were used to assess multicollinearity.

SmartPLS was used to estimate the partial least squares structural equation model (PLS-SEM). The measurement model was evaluated using Cronbach’s *α*, composite reliability (CR), average variance extracted (AVE), outer loadings, and discriminant validity. Following Hair et al. (31), *α* and CR values above 0.70, AVE above 0.50, outer loadings near or above 0.708, and heterotrait–monotrait (HTMT) ratios below 0.85 were used as reference criteria (32).

The structural model was evaluated using path coefficients, t values, *p* values, 95% confidence intervals, R^2^, f^2^, Q^2^, and the standardized root mean square residual (SRMR). Statistical significance was assessed with 5,000 bootstrap resamples. SRMR < 0.08 and Q^2^ > 0 were used as reference criteria (33). To prioritize the scientific findings in the main text, detailed distributional, collinearity, correlation, discriminant-validity, effect-size, fit, and predictive-relevance diagnostics are reported in Supplementary Tables S1–S8.

### 3.5 Ethics

This human-participant experiment received ethical approval from Qilu Institute of Technology, Jinan, Shandong, China. Participants provided electronic informed consent before random assignment. They were informed that participation was voluntary, that they could withdraw at any time, and that anonymized data would be used only for academic research. The disclosure stated that AI-generated content could not replace professional diagnosis or treatment. Participants were informed of the AI’s role before exposure and were debriefed after the experiment about the study purpose and the appropriate use of health information.

## 4 Results

### 4.1 Results overview

The analyses yielded three central findings. First, the randomized high-versus-low cue-package contrast produced a large difference in perceived anthropomorphic cues and increased social presence. Second, the primary structural paths from SP to SC, SC to TAHI, and TAHI to HIAI were positive and statistically significant. Third, the three prespecified indirect effects were significant, including the serial pathway from the cue-package contrast through SP and SC to TAHI. H1–H7 were therefore supported. The experimental inference applies to the overall package contrast on SP; the downstream paths and indirect effects are associations among post-exposure measures.

### 4.2 Sample characteristics

The final analytic sample included 536 participants, with 268 in each cue-package condition. Participants were predominantly younger and middle-aged: 174 (32.5%) were aged 18–25 years, 220 (41.0%) were aged 26–35 years, and 142 (26.5%) were aged 36 years or older. The sample included 240 men (44.8%) and 296 women (55.2%). Most participants (82.3%) reported at least occasional sleep disturbance during the previous month, and all reported at least some generative-AI use (Table 3).

**Table 3:** Demographic characteristics of respondents (*N* = 536).

Variable	Category	*n*	%
Gender	Male	240	44.8
	Female	296	55.2
Age	18–25 years	174	32.5
	26–35 years	220	41.0
	36 years and above	142	26.5
Education	Below high school	65	12.1
	Junior college	124	23.1
	Bachelor’s degree	275	51.3
	Graduate degree or above	72	13.4
Sleep disturbance	None	95	17.7
	Occasional	232	43.3
	Sometimes	140	26.1
	Almost every day	69	12.9
AI use frequency	High frequency (≥5 times/week)	174	32.5
	Often use (1–4 times/week)	214	39.9
	Occasional use (1–3 times/month)	108	20.1
	Rare use (<1 time/month)	40	7.5

Percentages may not sum to 100 because of rounding.

### 4.3 Manipulation check

This analysis assessed whether the two overall cue packages produced the intended difference in perceived anthropomorphic cues. Participants in the high-cue package reported higher scores (M = 5.239, SD = 0.779) than those in the low-cue package (M = 3.785, SD = 0.775), t(534) = 21.676, *p* < 0.001, Cohen’s d = 1.872 (Figure 4). The magnitude and direction of the difference indicate that the bundled manipulation was successful. The complete numeric summary is provided in Supplementary Table S2.

**Figure 4:**
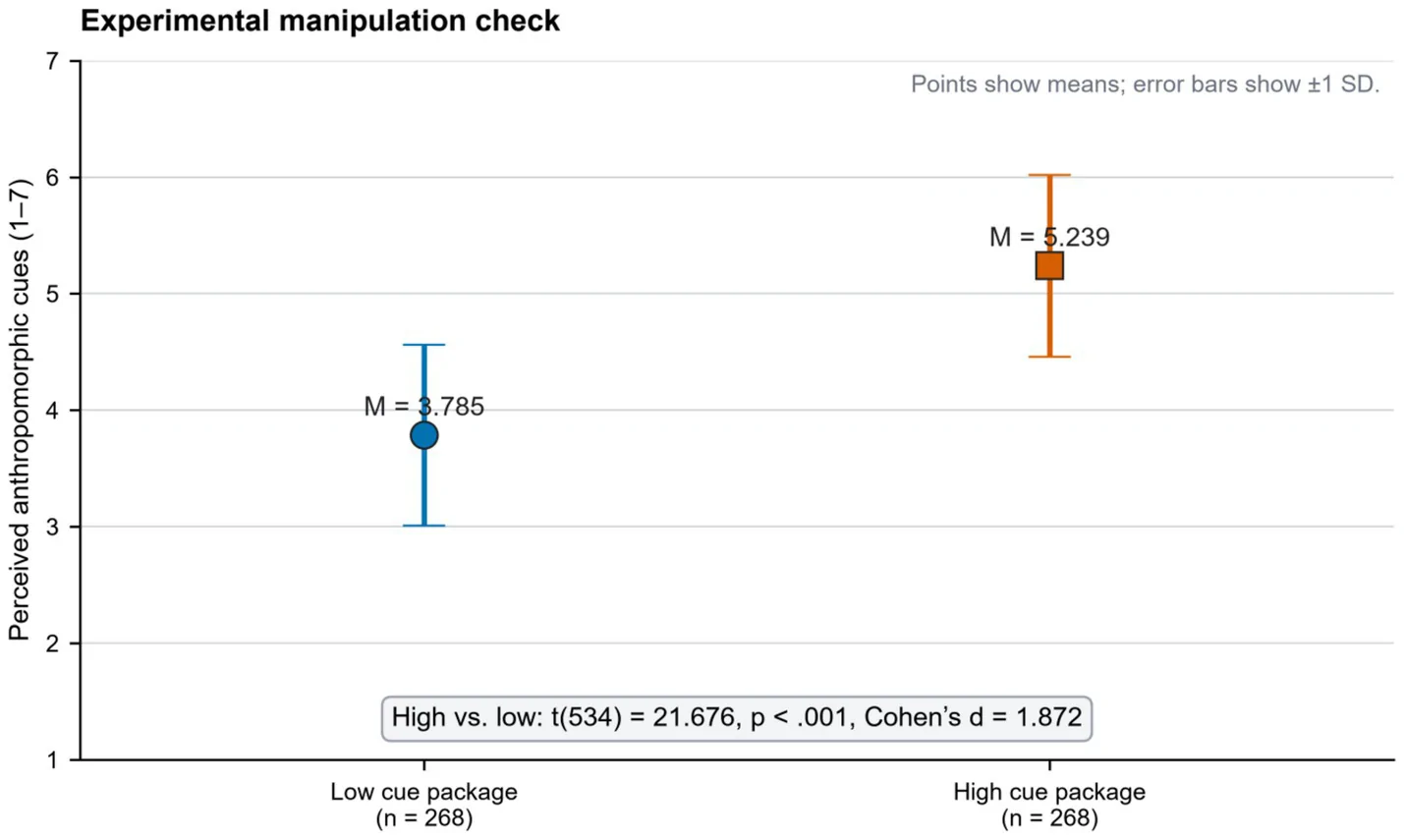
Experimental manipulation check for the overall cue packages. Points show group means and error bars show ±1 SD on the seven-point perceived-anthropomorphic-cues scale. The plotted means, SDs, and sample sizes reproduce the values in Supplementary Table S2. AC, anthropomorphic cues.

### 4.4 Measurement quality and preliminary diagnostics

Before hypothesis testing, the measurement model was evaluated for reliability, convergent validity, and discriminant validity. As summarized in Table 4, outer loadings ranged from 0.771 to 0.881, Cronbach’s α from 0.801 to 0.896, composite reliability from 0.883 to 0.923, and average variance extracted from 0.648 to 0.753. These values met the prespecified criteria. Complete Fitem-level distributional statistics, the full correlation matrix, HTMT ratios, and the Fornell–Larcker matrix are reported in Supplementary Tables S1, S4–S6.

**Table 4:** Construct descriptive statistics and measurement quality.

Construct	M	SD	Cronbach’s α	CR	AVE	Outer loading range
AC	4.52	1.07	0.830	0.899	0.748	0.858–0.875
SP	4.52	1.00	0.864	0.902	0.648	0.771–0.835
SC	4.49	1.04	0.896	0.923	0.706	0.807–0.860
TAHI	4.47	1.00	0.801	0.883	0.715	0.838–0.857
HIAI	4.47	1.09	0.835	0.901	0.753	0.849–0.881

AC = anthropomorphic cues; SP = social presence; SC = source credibility; TAHI = trust in AI-generated health information; HIAI = health information adoption intention; CR = composite reliability; AVE = average variance extracted.

The first unrotated factor accounted for 36.92% of the variance, and full-collinearity VIF values ranged from 1.126 to 1.788 (Supplementary Table S3). These screening results do not eliminate the possibility of common method variance because the focal mediators and outcomes were reported by the same participants at the same time.

### 4.5 Primary structural findings

This analysis tested H1–H3 and H7. The randomized cue-package contrast increased SP (β = 0.475, t = 11.659, *p* < 0.001, 95% CI [0.394, 0.554]), supporting H1. Among the post-exposure measures, SP was positively associated with SC (β = 0.483, t = 12.549, *p* < 0.001, 95% CI [0.408, 0.559]), SC was positively associated with TAHI (β = 0.508, t = 11.816, *p* < 0.001, 95% CI [0.424, 0.593]), and TAHI was positively associated with HIAI (β = 0.539, t = 13.770, *p* < 0.001, 95% CI [0.461, 0.614]). These estimates supported H2, H3, and H7 (Figure 5).

**Figure 5:**
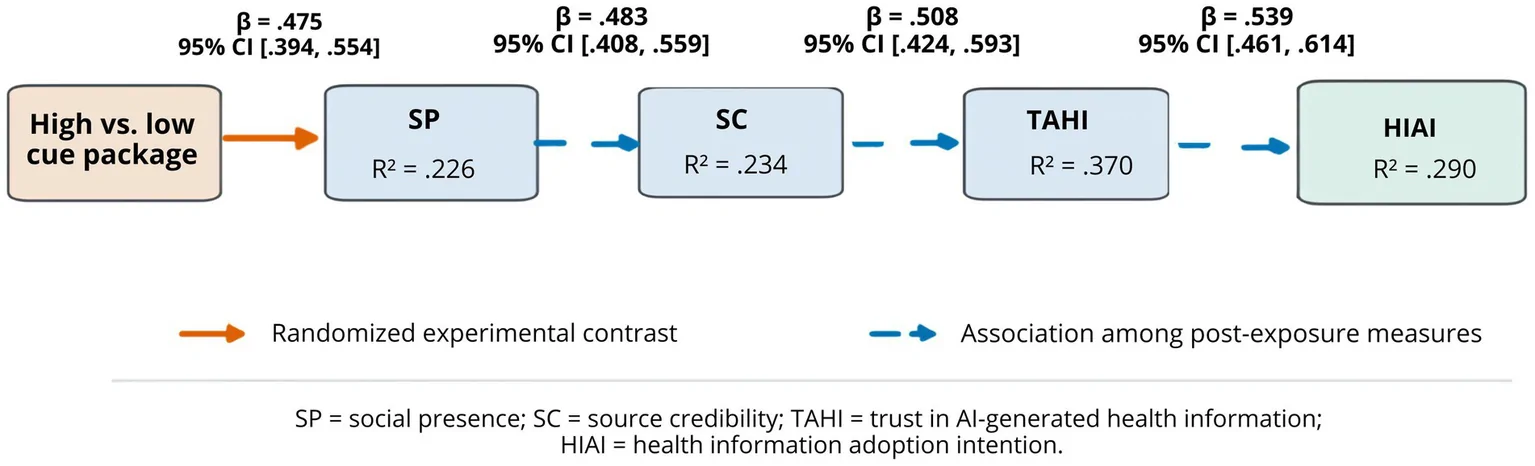
Primary structural-model findings. Path labels report standardized coefficients and 95% confidence intervals. R² values appear inside the endogenous constructs. The orange path represents the randomized high-versus-low cue-package contrast. Blue paths represent associations among simultaneously measured post-exposure constructs. All four displayed paths had *p* < 0.001. SP, social presence; SC, source credibility; TAHI, trust in AI-generated health information; HIAI, health information adoption intention.

The direct scientific implication is that the tested overall package altered participants’ immediate social-presence ratings, while higher ratings on each downstream construct were associated with higher ratings on the next construct in the hypothesized sequence. Only the first link was experimentally manipulated.

### 4.6 Indirect effects

This analysis tested H4–H6 using bootstrapped specific indirect effects. The cue-package contrast was indirectly associated with SC through SP (indirect effect = 0.398, *p* < 0.001, 95% CI [0.296, 0.512]), supporting H4. SP was indirectly associated with TAHI through SC (indirect effect = 0.245, *p* < 0.001, 95% CI [0.191, 0.306]), supporting H5. The serial indirect effect from the cue-package contrast through SP and SC to TAHI was also significant (indirect effect = 0.203, *p* < 0.01, 95% CI [0.143, 0.273]), supporting H6 (Figure 6).

**Figure 6:**
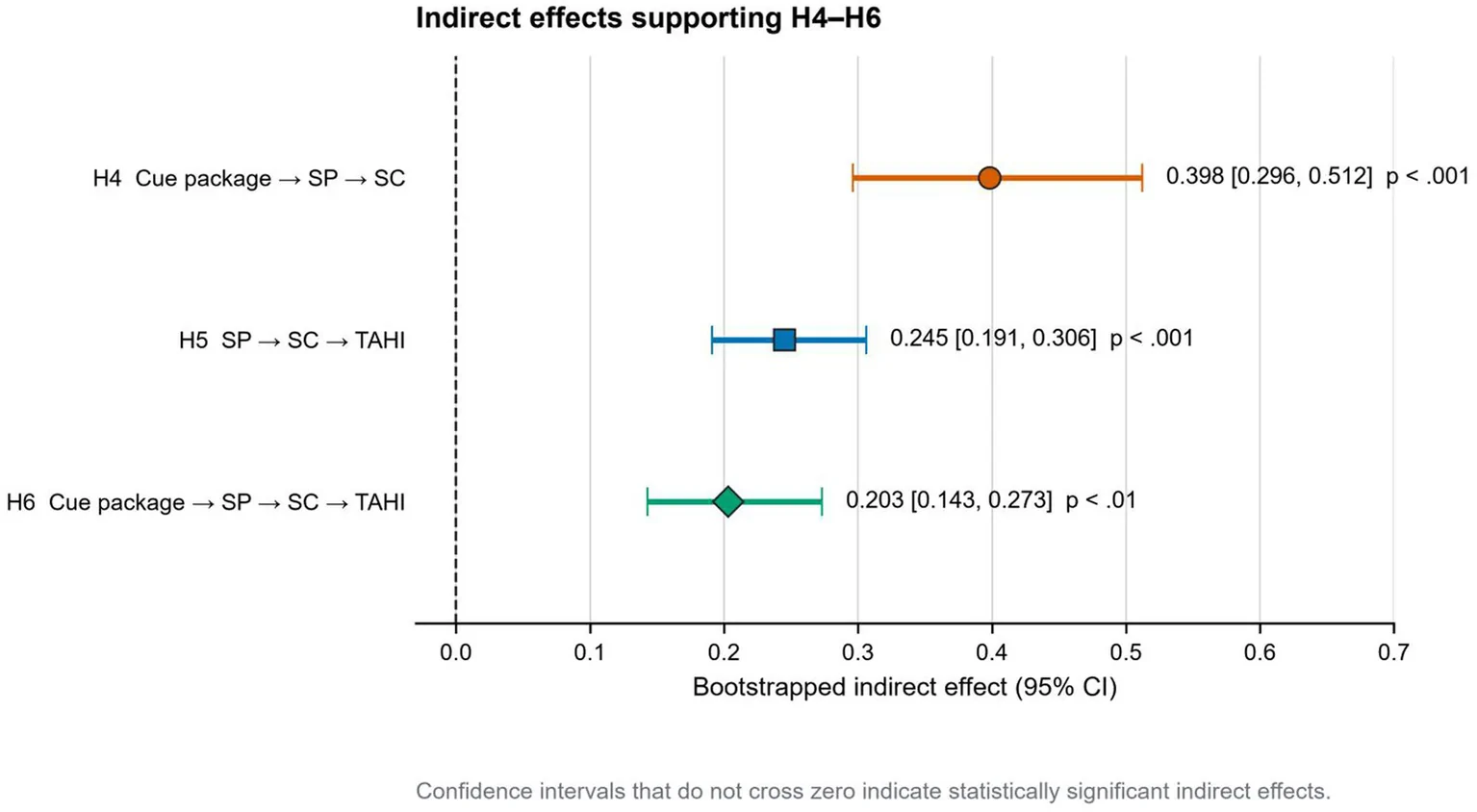
Bootstrapped indirect effects for H4–H6. Points show indirect-effect estimates and horizontal bars show the reported 95% confidence intervals. None of the intervals crosses zero. The figure uses the indirect-effect estimates from the original analysis without recalculation. SP, social presence; SC, source credibility; TAHI, trust in AI-generated health information.

For H4 and H5, the companion direct effects remained significant: 0.270, 95% CI [0.084, 0.454], and 0.170, 95% CI [0.089, 0.248], respectively. For H6, the direct effect of the cue-package contrast on TAHI was not significant (−0.041, 95% CI [−0.196, 0.114]), a pattern consistent with indirect-only mediation. These indirect effects support the hypothesized statistical pathways but do not independently establish temporal or causal order among SP, SC, and TAHI, which were measured at the same post-exposure occasion.

Table 5 consolidates the primary direct and indirect hypothesis tests so that effect estimates, uncertainty, significance, and hypothesis decisions can be reviewed in one place.

**Table 5:** Consolidated hypothesis tests.

Hypothesis	Tested path or effect	Statistical evidence	Decision
H1	Cue package → SP	β = 0.475; t = 11.659; *p* < 0.001; 95% CI [0.394, 0.554]	Supported
H2	SP → SC	β = 0.483; t = 12.549; *p* < 0.001; 95% CI [0.408, 0.559]	Supported
H3	SC → TAHI	β = 0.508; t = 11.816; *p* < 0.001; 95% CI [0.424, 0.593]	Supported
H4	Cue package → SP → SC	Indirect = 0.398; *p* < 0.001; 95% CI [0.296, 0.512]; direct = 0.270, 95% CI [0.084, 0.454]	Supported
H5	SP → SC → TAHI	Indirect = 0.245; *p* < 0.001; 95% CI [0.191, 0.306]; direct = 0.170, 95% CI [0.089, 0.248]	Supported
H6	Cue package → SP → SC → TAHI	Indirect = 0.203; *p* < 0.01; 95% CI [0.143, 0.273]; direct = −0.041, 95% CI [−0.196, 0.114]	Supported
H7	TAHI → HIAI	β = 0.539; t = 13.770; *p* < 0.001; 95% CI [0.461, 0.614]	Supported

The cue package was dummy coded 0 = low and 1 = high. β denotes a standardized path coefficient. Indirect-effect estimates are the bootstrapped values reported in the original analysis. H1 is supported by the randomized package contrast; H2, H3, and H7 concern structural associations; H4–H6 concern indirect effects among the specified constructs.

### 4.7 Structural diagnostics and interpretation boundaries

The model explained 22.6% of the variance in SP, 23.4% in SC, 37.0% in TAHI, and 29.0% in HIAI. The standardized root mean square residual was 0.056, and Stone–Geisser Q^2^ values were greater than zero for all endogenous constructs. Complete adjusted R^2^, f^2^, inner-VIF, SRMR, and Q^2^ results are provided in Supplementary Tables S7, S8. These diagnostics indicate partial explanatory coverage and acceptable predictive relevance under the prespecified criteria; they do not remove the design limitations associated with a bundled manipulation and same-time self-report mediators and outcomes.

## 5 Discussion

This study integrated CASA, Social Presence Theory, Source Credibility Theory, and the Information Adoption Model to examine an overall high-versus-low anthropomorphic-cue package in health communication. In a Chinese single-exposure experiment with explicit AI disclosure in both conditions, the package contrast increased initial SP. The post-exposure associations and indirect effects were consistent with the proposed sequence from SP to SC and TAHI, followed by an association between TAHI and HIAI. These findings concern short-term psychological responses to the tested packages, not component-specific effects, durable trust, or observed health behavior. The interpretation is limited to the sleep-related health-information context examined here.

### 5.1 Summary of findings

First, the overall high-versus-low anthropomorphic-cue package contrast increased initial social presence (H1). This finding is consistent with CASA, which proposes that people may apply social rules to computers and mediated entities (10, 34). Because the packages differed simultaneously in visual realism, gesture behavior, interpersonal/linguistic framing, and duration, the observed effect cannot be attributed to any one component. The inference is limited to the tested packages and this one exposure. Moreover, the study did not compare disclosed with undisclosed AI identity; it shows only that the overall package contrast operated within a fixed explicit-disclosure context.

Second, SP was positively associated with SC, and the bootstrapped estimates were consistent with SC statistically mediating the relationship between SP and TAHI (H2-H6). The findings distinguish relational experience from epistemic evaluation: warmth or co-presence did not by itself demonstrate professional competence, and participants also evaluated whether the VOL appeared knowledgeable, impartial, and honest (21, 35). Because SP, SC, and TAHI were measured at the same post-exposure time point, the observed ordering is theoretically specified and statistically compatible with the data but is not fully causally identified.

Third, TAHI was positively associated with HIAI (H7), consistent with the IAM (22). Participants reported stronger willingness to refer to, further verify, discuss, or consider the information when they rated the information as more trustworthy. This association should not be read as evidence that they subsequently clicked, shared, verified, consulted a clinician, adhered to advice, or changed a health behavior. The well-established intention-behavior gap makes objective follow-up necessary (23).

Overall, the anthropomorphic-cue package contrast did not have a significant direct effect on TAHI; instead, the serial indirect estimate through SP and SC was significant. This pattern is compatible with a staged pathway from the tested package contrast to social experience, source evaluation, and information trust, but it does not isolate the causal contribution of visual, gestural, linguistic, vocal, interactive, or duration components. The R-squared values show that the model explains meaningful but partial variance, and the present measures cannot exclude alternative explanations based on prior AI attitudes, e-health literacy, platform experience, algorithm aversion, AI anxiety, or health anxiety.

### 5.2 Theoretical and practical implications

#### 5.2.1 Theoretical implications

This study offers three theoretical contributions.

First, the study extends CASA to the intersection of AI virtual opinion leaders, health communication, and explicit AI identity disclosure. The present randomized contrast indicates that the tested overall high-versus-low anthropomorphic-cue package can increase initial social presence when AI identity is disclosed. Because disclosure was held constant, the study does not estimate whether disclosure itself increases, decreases, or moderates this effect; because the package bundled several differences, it also does not estimate the independent effect of any component (24, 26).

Second, the study differentiates SP as a relational experience, SC as an evaluation of epistemic competence and trustworthiness, and TAHI as trust in the factual reliability of a message (18, 35). The significant serial indirect estimate is compatible with a pathway in which social and cognitive evaluations both matter. This theoretical ordering is useful for future testing, but simultaneous measurement means that alternative temporal orders cannot be excluded by the present mediation analysis.

Third, integrating CASA, Social Presence Theory, Source Credibility Theory, and IAM yields a staged, non-exhaustive account of short-term AI health communication (14, 17, 22). The partial R-squared values and omitted individual differences indicate that the framework should be extended rather than treated as complete. E-health literacy, prior AI experience, algorithm aversion, AI anxiety, health anxiety, platform experience, and cultural norms are plausible covariates, moderators, or boundary conditions (36–38).

#### 5.2.2 Practical implications

First, developers should treat the tested manipulation as a bundled package of visual, behavioral, interpersonal/linguistic, and duration-related differences rather than infer that any one cue produced the observed effect. Within the tested contrast, the overall high-cue package increased initial SP; the experiment did not estimate component-specific effects or a continuous dose–response curve. Design recommendations should therefore be validated with cue-isolation or factorial experiments using equivalent content and duration before any specific visual, gestural, vocal, linguistic, or interactive feature is implemented as an evidence-based intervention.

Second, health organizations should maintain transparent AI identification while recognizing that disclosure was a fixed context rather than a tested treatment. The results do not show whether disclosure increases or decreases trust or changes the cue-package effect. A direct test would require a 2 × 2 Anthropomorphism × AI Disclosure design with high and low anthropomorphism crossed with disclosure and no-disclosure conditions, equivalent message content, and preregistered contrasts. Such a design could estimate both main effects and their interaction (24, 26).

Third, platforms should strengthen verifiable source cues and substantive information quality rather than rely on human likeness alone. Authoritative sources, transparent limitations, human review, and verifiable citations are defensible design principles, although these features were not manipulated here (19, 27). These implications concern perceived SP, SC, TAHI, and HIAI. The study does not establish that investment in anthropomorphic design produces actual behavior change, retention, clinical benefit, or a favorable return on cost; those questions require objective outcomes and cost-effectiveness evaluation.

Accordingly, within the present sleep-related health-information context, the practical interpretation is limited to willingness to consider information, trust-related psychological responses, and proximal adoption intention rather than actual adherence, health-behavior change, or clinical outcomes.

### 5.3 Limitations and future research

First, external validity is limited by the recruitment structure and final age distribution. After screening, 174 participants (32.5%) were aged 18–25 years, 220 (41.0%) were aged 26–35 years, and 142 (26.5%) were aged 36 years or older. The broad upper category was not designed as an older-adult stratum and does not show how many participants were aged 60 or 65 years or older. The online sample also reported at least some generative-AI use. Digital or e-health literacy was not measured. The study therefore cannot determine whether older adults, digitally disadvantaged groups, or AI-naive users would show the same expectations, trust judgments, or responses to anthropomorphic cues. Future research should use age-stratified sampling with a distinct older-adult group and recruit participants through community, clinical, assisted, and offline channels.

Second, the study was conducted in China and should be interpreted within the present Chinese sample and communication context. Cultural norms concerning health authority, interpersonal warmth, uncertainty, technology trust, and the acceptability of nonhuman social actors may change how AC is interpreted and how SP, SC, and TAHI are connected. Existing evidence indicates that culture can modulate anthropomorphism, although the direction is not uniform across studies (38). Cross-cultural replications and pre-registered multi-country comparisons should test measurement invariance before comparing path estimates.

Third, the structured, branched video provided experimental control but represented one short exposure. It did not reproduce repeated encounters, evolving parasocial expectations, error recovery, or trust calibration over weeks or months. Longitudinal panels, repeated-exposure experiments, and field studies on real health platforms should examine whether initial SP and SC persist, strengthen, or reverse over time.

Fourth, all focal mediators and outcomes were self-reported at the same time. HIAI is an intention measure and cannot be treated as actual verification, consultation, clinical follow-through, or health behavior (23). Health framing may also have encouraged socially desirable reports of prudent intentions. Future studies should combine anonymous platform logs and objective click, share, verification, retention, consultation, and follow-through measures with indirect measures or a validated social-desirability scale.

Fifth, the model did not measure e-health literacy, algorithm aversion, AI anxiety, health anxiety, prior AI attitudes, or platform experience. These omitted individual differences may explain part of the substantial variance not captured by the R-squared values and may operate as covariates, moderators, or boundary conditions (36, 37). The present model therefore cannot exclude these alternative explanations.

Sixth, AI disclosure was held constant across conditions, and the design lacked both a human-communicator control and a no-disclosure control. The study cannot estimate the independent or interactive effect of disclosure, compare AI VOLs with human communicators, or quantify the cue effect relative to these alternatives. A factorial design crossing human versus AI communicator, disclosure versus no disclosure, and low versus high anthropomorphism would provide direct tests. Similarly, the two tested AC conditions do not establish that higher anthropomorphism is always beneficial or that an inverted-U relationship exists; uncanny reactions, perceived manipulation, reactance, and AI anxiety remain plausible boundary conditions (5, 7, 39).

The cue manipulation bundled multiple dimensions rather than isolating individual components. The two videos differed in avatar realism, gesture frequency and variety, interpersonal/linguistic framing, and duration (approximately 150.64 s in the low package and 178.44 s in the high package). The present effect therefore belongs to the overall high-versus-low cue package; it cannot determine whether a visual, linguistic, vocal, behavioral, interactive, or duration-related component was independently responsible. Future cue-isolation, factorial, and component-specific manipulations should use equivalent message content and duration to separate these contributions.

Seventh, Harman’s test and the reported full-collinearity VIFs are limited diagnostics rather than proof that common method variance is absent. Same-source, same-time measurement may inflate associations among SP, SC, TAHI, and HIAI. Although random assignment supports causal inference for the overall cue-package contrast on SP, the mediators and outcomes were not experimentally manipulated and were measured at one post-exposure occasion. Reverse causality among these measured constructs and unmeasured confounding therefore cannot be ruled out. Future studies should use temporal separation, multi-source data, a theoretically appropriate marker variable, multi-wave longitudinal mediation, or experimental manipulation of the proposed mediators.

Eighth, all participants encountered sleep-related health information. Trust processes may differ across preventive versus treatment-related information and across topics with low versus high perceived severity or risk because authority expectations, verification needs, credibility standards, and willingness to rely on AI may change with context. The present data do not establish the direction of those differences. Replication should compare sleep advice with vaccination, medication, cancer-treatment, and emergency-care contexts and test whether topic severity or risk moderates the anthropomorphism effect.

## 6 Conclusion

This study examined how an overall high-versus-low anthropomorphic-cue package for AI virtual opinion leaders operates under a fixed explicit-AI-disclosure context. Using a single-factor randomized contrast and PLS-SEM with 536 valid responses, it provides contextualized evidence about initial psychological responses within the present Chinese sample rather than evidence about component-specific effects, long-term relationships, or population-wide effectiveness.

These conclusions apply first to the sleep-related health-information context studied here and should not be generalized to higher-risk medical topics without direct replication.

The overall high-versus-low anthropomorphic-cue package contrast increased initial SP. SP was positively associated with SC, SC was positively associated with TAHI, and TAHI was positively associated with HIAI. The direct effect of the package contrast on TAHI was not significant, whereas the bootstrapped serial indirect effect through SP and SC was significant. Random assignment supports the overall package contrast on SP; it does not identify which component produced the effect, and the remaining results provide evidence compatible with, but do not fully establish, the hypothesized causal sequence among simultaneously measured constructs.

The findings concern self-reported trust and adoption intention after one exposure, not actual behavior, durable trust, or a demonstrated advantage over human communicators. Disclosure was not manipulated, and the results do not establish that disclosure strengthened or weakened the AC effect. Replication is needed with older adults, digitally disadvantaged groups, culturally diverse samples, repeated and longitudinal exposure, real-world health platforms, human and disclosure controls, objective verification and click-through measures, clinician consultation, clinical follow-through, retention, health behavior, and cost-effectiveness outcomes.

## Data Availability

Due to the need to protect participant anonymity, the dataset cannot be made public. Requests to access the datasets should be directed to Shaohua Huo, huoshaohua1258@gmail.com.
