# Supplementary material for: High- versus low-anthropomorphic-cue packages and health-information trust: social presence and source credibility under explicit AI disclosure
Source: Front Public Health. 2026 Sep 30;14:1938022. doi: 10.3389/fpubh.2026.1938022 (PMC13662049; doi:10.3389/fpubh.2026.1938022)
Supplement: Supplementary file 1 [file Data_Sheet_1.PDF]

Supplementary Material

The following tables provide diagnostic and secondary statistical detail supporting the main-text results.

Table S1. Item-Level Normality Diagnostics

Items	M	SD	Skewness	Kurtosis
AC1	4.543	1.193	−0.069	−0.292
AC2	4.483	1.274	−0.063	−0.325
AC3	4.509	1.195	−0.137	−0.307
SP1	4.519	1.178	−0.244	0.023
SP2	4.560	1.218	0.026	−0.483
SP3	4.489	1.212	−0.049	−0.413
SP4	4.494	1.233	−0.083	−0.337
SP5	4.494	1.233	−0.029	−0.317
SC1	4.487	1.184	−0.212	−0.207
SC2	4.494	1.243	−0.075	−0.428
SC3	4.472	1.166	−0.191	−0.026
SC4	4.470	1.245	−0.112	−0.399
SC5	4.534	1.209	−0.091	−0.152
TAHI1	4.498	1.266	−0.123	−0.330
TAHI2	4.403	1.193	−0.193	−0.143
TAHI3	4.543	1.190	0.031	−0.400
HIAI1	4.528	1.210	−0.183	−0.114
HIAI2	4.474	1.198	−0.138	−0.036
HIAI3	4.496	1.218	−0.252	−0.123

Note. AC = anthropomorphic cues; SP = social presence; SC = source credibility; TAHI = trust in AI-generated health information; HIAI = health information adoption intention.

Table S2. Manipulation Check Results

Group	*n*	M	SD	t(df)	*p*	Cohen's d
High AC	268	5.239	0.779	21.676 (534)	< .001	1.872
Low AC	268	3.785	0.775			

Note. The dependent variable is the composite score of perceived anthropomorphic cues (AC).

Table S3. Full-Collinearity VIF Test

Construct	AC	SP	SC	TAHI	HIAI
Full collinearity VIF	1.126	1.599	1.788	1.768	1.408

Note. VIF = variance inflation factor.

Table S4. Descriptive Statistics and Correlations

Construct	M	SD	1	2	3	4	5
1. AC	4.52	1.07	1				
2. SP	4.52	1.00	.357***	1			
3. SC	4.49	1.04	.242***	.483***	1		
4. TAHI	4.47	1.00	.199***	.416***	.590***	1	
5. HIAI	4.47	1.09	.161***	.253***	.356***	.539***	1

Note. * $p < .05$; ** $p < .01$; *** $p < .001$; AC = anthropomorphic cues; SP = social presence; SC = source credibility; TAHI = trust in AI-generated health information; HIAI = health information adoption intention.

Table S5. Heterotrait–Monotrait Ratios

Construct	AC	SP	SC	TAHI
SP	0.387			
SC	0.186	0.607		
TAHI	0.131	0.497	0.650	
HIAI	0.071	0.257	0.468	0.627

Note. AC = anthropomorphic cues; SP = social presence; SC = source credibility; TAHI = trust in AI-generated health information; HIAI = health information adoption intention.

Table S6. Fornell–Larcker Criterion

Construct	AC	SP	SC	TAHI	HIAI
AC	**0.865**				
SP	0.357	**0.805**			
SC	0.242	0.483	**0.840**		
TAHI	0.199	0.416	0.590	**0.846**	
HIAI	0.161	0.253	0.356	0.539	**0.868**

Note. Diagonal values are square roots of AVE. AC = anthropomorphic cues; SP = social presence; SC = source credibility; TAHI = trust in AI-generated health information; HIAI = health information adoption intention.

Table S7. Explained Variance, Effect Sizes, and Inner VIF

Construct	R^2	R^2adj	Path	f^2	Inner VIF
SP	0.226	0.225	AC → SP	0.292	1.000
SC	0.234	0.232	SP → SC	0.305	1.000
TAHI	0.370	0.367	SP → TAHI	0.036	1.305
			SC → TAHI	0.314	1.305
HIAI	0.290	0.289	TAHI → HIAI	0.408	1.000

Note. AC = anthropomorphic cues (0 = low cue package, 1 = high cue package); SP = social presence; SC = source credibility; TAHI = trust in AI-generated health information; HIAI = health information adoption intention.

Table S8. Model Fit and Stone–Geisser Q^2

Index	SP	SC	TAHI	HIAI	Criterion
Q^2	0.173	0.206	0.245	0.199	> 0
SRMR	0.056				< 0.08

Note. SRMR = standardized root mean square residual; SP = social presence; SC = source credibility; TAHI = trust in AI-generated health information; HIAI = health information adoption intention.
